# Revisiting the hybridization processes in the *Triatoma brasiliensis* complex (Hemiptera, Triatominae): Interspecific genomic compatibility point to a possible recent diversification of the species grouped in this monophyletic complex

**DOI:** 10.1371/journal.pone.0257992

**Published:** 2021-10-15

**Authors:** Heloisa Pinotti, Jader de Oliveira, Amanda Ravazi, Fernanda Fernandez Madeira, Yago Visinho dos Reis, Ana Beatriz Bortolozo de Oliveira, Maria Tercília Vilela de Azeredo-Oliveira, João Aristeu da Rosa, Kaio Cesar Chaboli Alevi

**Affiliations:** 1 Laboratório de Parasitologia, Departamento de Ciências Biológicas, Universidade Estadual Paulista “Júlio de Mesquita Filho” (UNESP), Faculdade de Ciências Farmacêuticas, Araraquara, São Paulo, Brasil; 2 Instituto de Biociências, Universidade Estadual Paulista “Júlio de Mesquita Filho”, IBB/UNESP, Botucatu, São Paulo, Brasil; 3 Laboratório de Biologia Celular, Departamento de Biologia, Instituto de Biociências, Letras e Ciências Exatas, Universidade Estadual Paulista “Júlio de Mesquita Filho”, IBILCE/UNESP, São José do Rio Preto, São Paulo, Brasil; Texas A&M University College of Veterinary Medicine, UNITED STATES

## Abstract

Triatomines are hematophagous insects of great epidemiological importance, since they are vectors of the protozoan *Trypanosoma cruzi*, the etiological agent of Chagas disease. *Triatoma brasiliensis* complex is a monophyletic group formed by two subspecies and six species: *T*. *b*. *brasiliensis*, *T*. *b*. *macromelasoma*, *T*. *bahiensis*, *T*. *juazeirensis*, *T*. *lenti*, *T*. *melanica*, *T*. *petrocchiae* and *T*. *sherlocki*. The specific status of several species grouped in the *T*. *brasiliensis* complex was confirmed from experimental crossing and analysis of reproductive barriers. Thus, we perform interspecific experimental crosses between *T*. *lenti* and other species and subspecies of the *T*. *brasiliensis* complex and perform morphological analysis of the gonads and cytogenetic analysis in the homeologous chromosomes of the hybrids of first generation (F1). Besides that, we rescue all the literature data associated with the study of reproductive barriers in this monophyletic complex of species and subspecies. For all crosses performed between *T*. *b*. *brasiliensis*, *T*. *b*. *macromelasoma*, *T*. *juazeirensis* and *T*. *melanica* with *T*. *lenti*, interspecific copulas occurred (showing absence of mechanical isolation), hybrids were obtained, none of the male hybrids presented the phenomenon of gonadal dysgenesis and 100% pairing between the chromosomes homeologous of the hybrids was observed. Thus, we demonstrate that there are no pre-zygotic reproductive barriers installed between *T*. *lenti* and the species and subspecies of the *T*. *brasiliensis* complex. In addition, we demonstrate that the hybrids obtained between these crosses have high genomic compatibility and the absence of gonadal dysgenesis. These results point to reproductive compatibility between *T*. *lenti* and species and subspecies of the *T*. *brasiliensis* complex (confirming its inclusion in the complex) and lead us to suggest a possible recent diversification of the taxa of this monophyletic group.

## Introduction

Triatomines are hematophagous insects of great epidemiological importance, since they are vectors of the protozoan *Trypanosoma cruzi* (Chagas, 1909) (Kinetoplastida, Trypanosomatidae), the etiological agent of Chagas disease [1]. This disease is neglected, has no cure in the chronic phase, affects about eight million people and puts at risk of infection, approximately, 25 million people worldwide [1]. Cases of cure from the use of the drugs Benznidazole or Nifurtimox are reported only when the diagnosis is made in the acute phase of the infection (usually asymptomatic) [1]. Thus, the control of vector populations is considered the main way to minimize the incidence of new chagasic cases [1], being the studies related to triatomines of extreme importance for public health, since they can generate subsidies to assist these programs in the prophylaxis of Chagas disease.

There are currently 157 species of triatomines, grouped into 18 genera and five tribes [2–5]. Based mainly on morphological characters and/or geographic distribution, triatomines were grouped into complexes and subcomplexes [6–10]. Although these groupings are not valid according to the International Code of Zoological Nomenclature [11], Justi et al. [12] emphasize that complexes and subcomplexes of species must form natural groups (monophyletic groups), which led to several changes in the initial groupings [12–19].

*Triatoma brasiliensis* complex is a group of species and subspecies that has its center of dispersion in the semi-arid region of Northeast Brazil [20]. This grouping of species and subspecies was initially proposed by Lucena [7] as a systematic arrangement composed mainly of taxa found in the Northeast region of Brazil: *T*. *b*. *brasiliensis* Neiva, 1911, *T*. *petrocchiae* Pinto & Barreto, 1925, *T*. *lenti* Sherlock & Serafim, 1967, *T*. *pessoai* Sherlock and Serafim, 1967 (which was later synonymous with *T*. *lenti* [8]), *T*. *bahiensis* Sherlock & Serafim, 1967 (which was later synonymous with *T*. *lenti* [8]), *T*. *b*. *melanica* Neiva & Lent, 1941 (which was later synonymous with *T*. *b*. *brasiliensis* [8]) and *T*. *b*. *macromelasoma* Galvão, 1956 (which was also synonymous with *T*. *b*. *brasiliensis* [8]). Multidisciplinary studies based on morphology [21–23], biology [24], crossing experiments [18, 25–29], ecology [30, 31], isoenzymes [32], dispersal abilities [33], cytogenetics [34–38] and phylogenetics [22, 28, 39–41] allowed to demonstrate that *T*. *b*. *macromelasoma* and *T*. *b*. *melanica* were different taxa from *T*. *b*. *brasiliensis* (*T*. *b*. *macromelasoma* was redescribed and revalidated [42] and the status of *T*. *b*. *melanica* was raised to *T*. *melanica* [43]), allowed to describe the distinct population of *T*. *brasiliensis* from Juazeiro, Bahia, Brazil as *T*. *juazeirensis* Costa & Félix, 2007 [44], as well as allowed to revalidate the specific status of *T*. *bahiensis* [28], demonstrating that the *T*. *brasiliensis* complex is a monophyletic group formed by two subspecies and six species: *T*. *b*. *brasiliensis*, *T*. *b*. *macromelasoma*, *T*. *bahiensis*, *T*. *juazeirensis*, *T*. *lenti*, *T*. *melanica*, *T*. *petrocchiae* and *T*. *sherlocki* Papa et al. 2002 [22, 28, 39–41, 45].

*Triatoma b*. *brasiliensis* is one of the main Chagas disease vectors in the Northeast region of Brazil [46], as it is directly related to the semi-arid climate of this region [47–50]. This subspecies was reported in five Brazilian states: Maranhão, Piauí, Ceará, Rio Grande do Norte and Paraíba [31]. Until 2007, *T*. *juazeirensis* was considered as a population of *T*. *b*. *brasiliensis* collected in Juazeiro, Bahia, Brazil [44], being this species distributed in the states of Bahia and Pernambuco [31, 41]. In addition, *T*. *melanica*, until 2006, was considered as a subspecies of *T*. *b*. *brasiliensis* [43] endemic to the state of Minas Gerais and south of Bahia [31, 51]. *Triatoma petrocchiae* is a species morphologically related to *T*. *b*. *brasiliensis* [7] that is distributed in the states of Bahia, Ceará, Paraíba, Pernambuco and Rio Grande do Norte [31, 51]. *Triatoma lenti* and *T*. *bahiensis* were described, in 1967, as melanic forms of *T*. *b*. *brasiliensis* [52]. For 37 years, *T*. *bahiensis* was considered synonymous with *T*. *lenti* [8] and, recently, specimens were collected in the state of Bahia and the species was revalidated [28]. *Triatoma lenti* was notified in two Brazilian states, namely, Bahia and Goiás [31, 51, 53]. *Triatoma sherlocki* is an endemic species from Bahia [50, 54]. Finally, the subspecies *T*. *b*. *macromelasoma*, described based on melanic forms of *T*. *b*. *brasiliensis* found at home in the state of Pernambuco [31, 51], was redescribed and revalidated by Costa et al. [42].

Evolutionary studies, based on interspecific experimental crossings contribute to the taxonomy and systematics of triatomines (considering the biological concept of species proposed by Mayr [55, 56] and Dobzhansky [57]) [6, 18, 58]. The specific status of species grouped in the *T*. *brasiliensis* complex (such as *T*. *petrocchiae* [59], *T*. *sherlocki* [27], *T*. *lenti* [27] and *T*. *bahiensis* [28, 29]), for example, was confirmed from experimental crossing and analysis of pre-zygotic and post-zygotic reproductive barriers.

Thus, taking into account that the study of experimental crosses and of the resulting hybrids can (a) help to understand the systematics of this vectors complex, (b) can be used to analyze the isolation mechanisms that limit gene flow between the different species, (c) as well as can be used to establish the role of natural hybridization in the generation of new variants (which can lead to adaptive evolution and/or the foundation of new evolutionary lineages) [58, 60], we performed interspecific experimental crosses between *T*. *lenti* and other species and subspecies of the *T*. *brasiliensis* complex and perform morphological analysis of the gonads (with emphasis in the gonadal dysgenesis) and cytogenetic analysis in the homeologous chromosomes of the hybrids of first generation (F1) (with emphasis in the interspecific genomic compatibility). Besides that, we rescue all the literature data associated with the study of reproductive barriers in this monophyletic complex.

## Methods

Reciprocal experimental crosses were conducted between *T*. *b*. *brasiliensis*, *T*. *b*. *macromelasoma*, *T*. *juazeirensis* and *T*. *melanica* with *T*. *lenti* (Table 1) to assess whether there is an interspecific pre-zygotic reproductive barrier (phenomena that prevent copulation or interspecific fertilization) [55–57] installed between these species and subspecies of the *T*. *brasiliensis* complex. The insects used in the experiment came from colonies kept in the Triatominae insectary of the School of Pharmaceutical Sciences, São Paulo State University (UNESP), Araraquara, São Paulo, Brazil. These species and subspecies were identified with the help of the dichotomous keys developed by Costa et al. [42] and Dale et al. [51]. The experimental crosses were conducted in the Triatominae insectary of the School of Pharmaceutical Sciences, according to the experiments of Costa et al. [25] and Mendonça et al. [27]: the insects were sexed as 5th instar nymphs, and males and females were kept separately until they reached the adult stage in order to guarantee the virginity of the insects used in the crosses. For the experimental crosses, three couples from each set were placed in plastic jars (diameter 5 cm × height 10 cm) and kept at room temperature. Furthermore, intraspecific crosses were also performed for group control (Table 1). The eggs were collected weekly throughout the female’s oviposition periods and the egg fertility rate was calculated (Table 1).

**Table 1:** Experimental crosses performed between *T*. *b*. *brasiliensis*, *T*. *b*. *macromelasoma*, *T*. *juazeirensis* and *T*. *melanica* with *T*. *lenti*.

Experimental crosses	Number of eggs				Egg viability
	**C1**	**C2**	**C3**	**Total**	
*T*. *b*. *brasiliensis* ♀ x *T*. *lenti* ♂	100	84	93	277	49 (17.7%)
*T*. *lenti* ♀ x *T*. *b*. *brasiliensis* ♂	63	53	29	145	07 (4.8%)
*T*. *b*. *macromelasoma* ♀ x *T*. *lenti* ♂	58	34	19	111	23 (20.7%)
*T*. *lenti* ♀ x *T*. *b*. *macromelasoma* ♂	100	20	59	179	52 (29%)
*T*. *lenti* ♀ x *T*. *juazeirensis* ♂	34	108	104	246	73 (29.7%)
*T*. *juazeirensis* ♀ x *T*. *lenti* ♂	60	07	43	110	53 (48.2%)
*T*. *lenti* ♀ x *T*. *melanica* ♂	19	07	36	62	26 (41.9%)
*T*. *melanica* ♂ x *T*. *lenti* ♂	00	38	32	70	23 (32.8%)
**Control experiments**	**C1**	**C2**	**C3**	**Total**	
*T*. *b*. *brasiliensis* ♀ x *T*. *b*. *brasiliensis* ♂	79	192	-	271	159 (58.7%)
*T*. *melanica* ♀ x *T*. *melanica* ♂	25	111	-	136	129 (94.8%)
*T*. *lenti* ♀ x *T*. *lenti* ♂	75	104		179	103 (57.5%)
*T*. *b*. *macromelasoma* ♀ x *T*. *b*. *macromelasoma* ♂	90	84		174	108 (62.1%)
*T*. *juazeirensis* ♀ x *T*. *juazeirensis* ♂	-	-	-	-	163^1^

^1^ Costa et al. [25].

The gonads morphology of at least two adult male hybrids F1 from each cross (Table 1) was analyzed using a Leica MZ APO stereoscope microscope with Motic Advanced 3.2 plus image analysis system to assess the presence of the phenomenon of gonadal dysgenesis (which can be uni or bilateral) [61] and, posteriorly, cytogenetic analysis with Lacto-Acetic Orcein (De Vaio et al. [62], with modifications according to Alevi et al. [14]) were performed on slides prepared with these gonads and examined using Jenaval light microscopy (Zeiss), coupled to the digital camera and the Axio Vision LE 4.8 image analyzer system (Copyright © 2006–2009 Carl Zeiss Imaging Solutions Gmb H) to assess the degree of pairing between homeologous chromosomes (as established by Mendonça et al. [27]). Lastly, all data of the literature referring to experimental crossing studies in the *T*. *brasiliensis* complex were compiled (Table 2), with emphasis in the analysis of the presence/absence of pre-zygotic reproductive barrier.

**Table 2:** Experimental crosses performed between *T*. *brasiliensis* species complex.

Experimental crosses	Egg viability	Pre-zygotic isolation	References
**Interespecíficos**			
*T*. *b*. *brasiliensis* ♀ x *T*. *lenti* ♂	136 (51.1%)	Absent	[79]
*T*. *b*. *brasiliensis* ♀ x *T*. *lenti* ♂	49 (17.7%)	Absent	This paper
*T*. *lenti* ♀ x *T*. *b*. *brasiliensis* ♂	17 (100%)	Absent	[79]
*T*. *lenti* ♀ x *T*. *b*. *brasiliensis* ♂	7 (4.8%)	Absent	This paper
*T*. *b*. *brasiliensis* ♀ x *T*. *melanica* ♂	167^1^	Absent	[25]
*T*. *melanica* ♀ x *T*. *b*. *brasiliensis* ♂	132^1^	Absent	[25]
*T*. *b*. *macromelasoma* ♀ x *T*. *melanica* ♂	125^1^	Absent	[25]
*T*. *melanica* ♀ x *T*. *b*. *macromelasoma* ♂	94^1^	Absent	[25]
*T*. *b*. *brasiliensis* ♀ x *T*. *b*. *macromelasoma* ♂	128^1^	Absent	[25]
*T*. *b*. *macromelasoma* ♀ x *T*. *b*. *brasiliensis* ♂	181^1^	Absent	[25]
*T*. *b*. *macromelasoma* ♀ x *T*. *lenti* ♂	23 (20.7%)	Absent	This paper
*T*. *lenti* ♀ x *T*. *b*. *macromelasoma* ♂	52 (29%)	Absent	This paper
*T*. *b*. *brasiliensis* ♀ x *T*. *juazeirensis* ♂	228^1^	Absent	[25]
*T*. *juazeirensis* ♀ x *T*. *b*. *brasiliensis* ♂	195^1^	Absent	[25]
*T*. *lenti* ♀ x *T*. *juazeirensis* ♂	73 (29.7%)	Absent	This paper
*T*. *juazeirensis* ♀ x *T*. *lenti* ♂	53 (48.2%)	Absent	This paper
*T*. *juazeirensis* ♀ x *T*. *melanica* ♂	190^1^	Absent	[25]
*T*. *melanica* ♀ x *T*. *juazeirensis* ♂	81^1^	Absent	[25]
*T*. *b*. *macromelasoma* ♀ x *T*. *juazeirensis* ♂	208^1^	Absent	[25]
*T*. *juazeirensis* ♀ x *T*. *b*. *macromelasoma* ♂	60^1^	Absent	[25]
*T*. *juazeirensis* ♀ x *T*. *sherlocki* ♂	120 (73%)	Absent	[26]
*T*. *sherlocki* ♀ x *T*. *juazeirensis* ♂	68 (55%)	Absent	[26]
*T*. *sherlocki* ♀ x *T*. *b*. *brasiliensis* ♂	68 (54%)	Absent	[26]
*T*. *b*. *brasiliensis* ♀ x *T*. *sherlocki* ♂	68 (67%)	Absent	[26]
*T*. *sherlocki* ♀ x *T*. *melanica* ♂	23 (35%)	Absent	[26]
*T*. *lenti* ♀ x *T*. *melanica* ♂	26 (41.9%)	Absent	This paper
*T*. *melanica* ♂ x *T*. *lenti* ♂	23 (32.8%)	Absent	This paper
*T*. *sherlocki* ♀ x *T*. *lenti* ♂	65%	Absent	[27]
*T*. *lenti* ♀ x *T*. *sherlocki* ♂	7%	Absent	[27]
*T*. *bahiensis* ♀ x *T*. *lenti* ♂	70 (25.8%)	Absent	[28]
*T*. *lenti* ♀ x *T*. *bahiensis* ♂	84 (35.1%)	Absent	[28]
*T*. *b*. *brasiliensis* ♀ x *T*. *petrocchiae* ♂	0 (0%)	Present	[59]
*T*. *petrocchiae* ♀ x *T*. *b*. *brasiliensis* ♂	1 (1.4%)	Absent	[59]

^1^ Number of hybrids that hatched and reached adulthood.

## Results and discussion

The first experimental crossings performed with species and subspecies of the *T*. *brasiliensis* complex were between *T*. *petrocchiae* and *T*. *b*. *brasiliensis* [59]. Posteriorly, Costa et al. [25], Almeida et al. [33], Correia et al. [26] and Mendonça et al. [27] performed crossings between different populations of *T*. *b*. *brasiliensis* [20] and between *T*. *sherlocki* and members of the *T*. *brasiliensis* complex [26, 27, 33], respectively, and observed reproductive compatibility between the species and subspecies of the complex. Finally, Neves et al. [18] performed a cross between *T*. *melanocephala* Neiva and Pinto (1923), *T*. *tibiamaculata* (Pinto, 1926) and *T*. *vitticeps* (Stal, 1859) with *T*. *b*. *brasiliensis* and confirmed that these species are unrelated to the *T*. *brasiliensis* complex (as initially suggested by Schofield and Galvão [10]).

**Fig 1:**
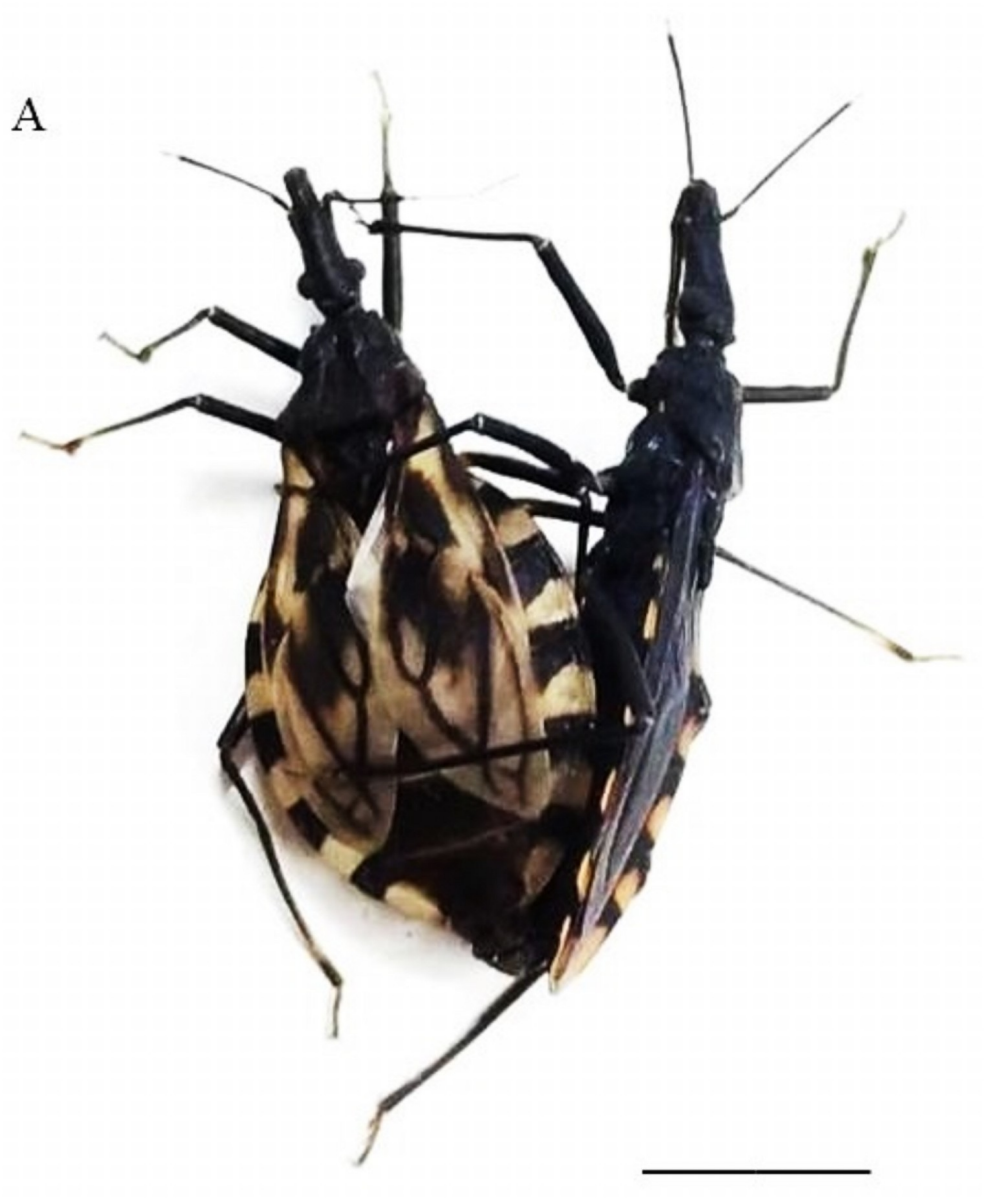
Example of interspecific copulation observed among species of the *T*. *brasiliensis* complex: *T*. *juazeirensis* female x *T*. *lenti* male. Bar: 5 cm.

**Fig 2:**
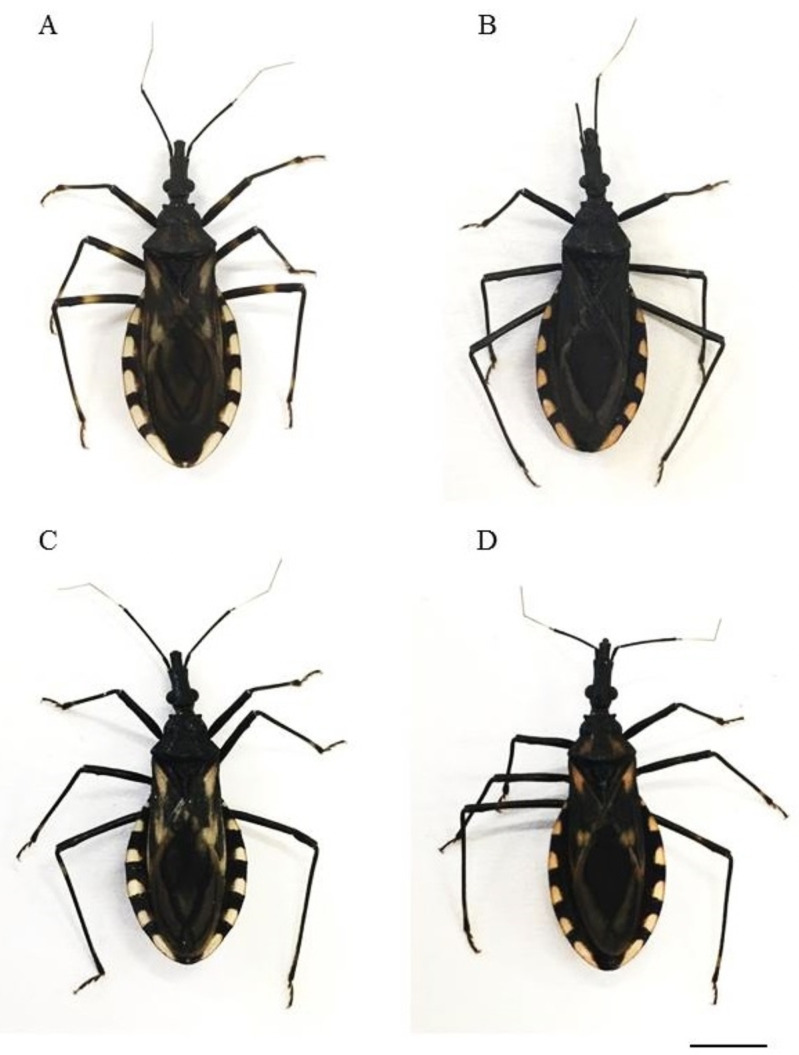
Hybrids resulting from experimental crosses between *T*. *b*. *brasiliensis* female x *T*. *lenti* male (A), *T*. *b*. *macromelasoma* female x *T*. *lenti* male (B), *T*. *juazeirensis* female x *T*. *lenti* male (C) and *T*. *melanica* female x *T*. *lenti* male (D). Bar: 5 cm.

For all crosses performed (Table 1), interspecific copulas occurred (Fig 1) [showing absence of mechanical isolation] and hybrids were obtained (Fig 2). Mechanical isolation, a pre-zygotic reproductive barrier characterized by incompatibility of the genitals [55, 56], has been reported several times in the subfamily Triatominae (acting in one or both directions of the crossings) [6, 63]. This reproductive compatibility observed between *T*. *lenti* and the species and subspecies of the *T*. *brasiliensis* complex confirms the inclusion of the species in the complex (as well as Correia et al. [26] confirmed the relationship of *T*. *sherlocki* with the *T*. *brasiliensis* complex).

Although hybrids were obtained for all directions of the crosses (Table 1), the hatch rates varied greatly, being the lowest rate observed for the crossing between *T*. *b*. *brasiliensis* and *T*. *lenti* (both directions) and the highest rates related to crosses between *T*. *juazeirensis* female and *T*. *lenti* male and between *T*. *melanica* and *T*. *lenti* (both directions). The only species of the *T*. *brasiliensis* complex that present genetic distance very close to the minimum stipulated to consider a species as valid are *T*. *melanica* and *T*. *lenti* [28]. However, it is not possible to correlate the low interspecific distance with the hatching rate because the genetic distance between *T*. *juazeirensis* and *T*. *lenti* is higher than 10% [28] (close to the observed distance between *T*. *lenti* and *T*. *infestans* [28] which are phylogenetically distant species [64] and do not produce hybrids [65]).

Justi et al. [64] suggest that climate change resulting from the Andean elevation [which occurred approximately 14 million years ago (Mya)] are associated with allopatric events responsible for the origin of the *T*. *brasiliensis* complex in the Caatinga biome, as well as the other complexes present in several biomes in the Latin America: *T*. *rubrovaria* in the Pampa, *T*. *infestans* in the Chaco and *T*. *sordida*, *T*. *matogrossensis* and part of *T*. *maculata* in the Cerrado. *Triatoma b*. *brasiliensis* when crossed with species of the *T*. *sordida* [65], *T*. *infestans* [65] and *T*. *vitticeps* complexes [18] do not result in fertile eggs. On the other hand, considering the biological concept of species [55–57], the absence of pre-zygotic reproductive barriers among practically all species and subspecies of the *T*. *brasiliensis* complex (Table 2) indicates that the taxa of this group may have diverged recently (being necessary the action of post-zygotic barriers—unfeasibility, sterility or collapse of the hybrid [56, 57]—to break the hybrids in nature).

Patterson and Gaunt [66] demonstrate that triatomines have diversified 95 Mya, putatively linking the origin of haematophagous behavior to the origin of South America. Monteiro et al. [39] performed an estimate of the divergence time associated with the speciation of the members of the *T*. *brasiliensis* complex (with emphasis on the relationship between *T*. *b*. *brasiliensis* and *T*. *melanica*) and suggested 5.2 Myr of independent evolution between these taxa. Considering the origin of Triatominae [66], the 5.2 Myr dating confirms the recent diversification of the species and subspecies of the *T*. *brasiliensis* complex.

**Fig 3:**
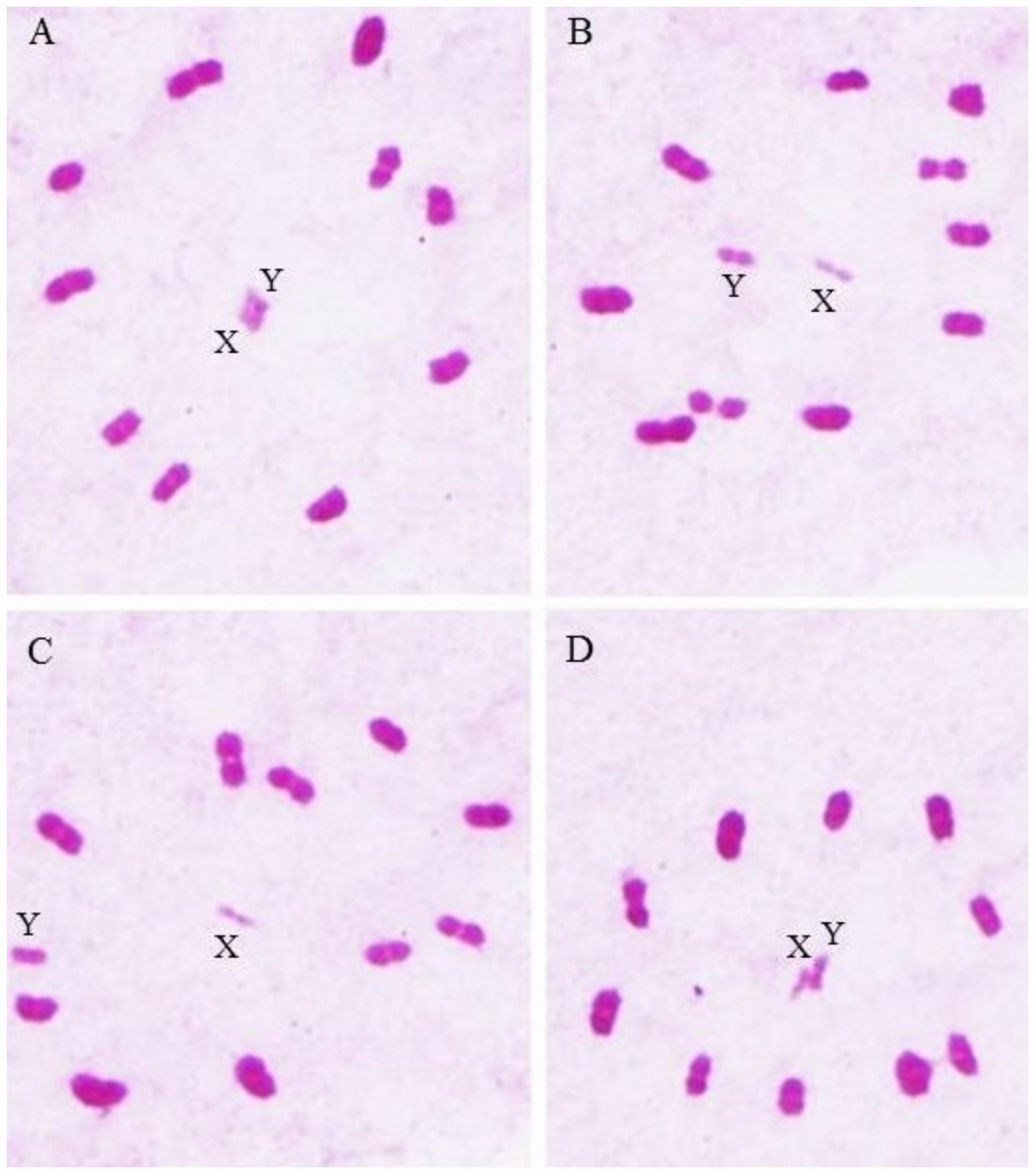
Metaphases I of hybrids from the cross between species of the *T*. *brasiliensis* complex: *T*. *b*. *brasiliensis* female x *T*. *lenti* male (A), *T*. *b*. *macromelasoma* female x *T*. *lenti* male (B), *T*. *juazeirensis* female x *T*. *lenti* male (C) and *T*. *melanica* female x *T*. *lenti* male (D). Note that 100% of the chromosomes were paired. X: X sex chromosome; Y: Y sex chromosome; Bar: 10 μm.

Taking into account that to occur pairing between the genome of different species there must be at least 80% homeology between chromosomes (which allows to evaluate the evolutionary relationship of the species) [67], we cytogenetically analyzed the genomic compatibility of the *T*. *brasiliensis* species and subspecies complex, through the degree of pairing of the homeologous chromosomes of the hybrids in metaphase I. The results obtained demonstrated 100% pairing between the chromosomes of the hybrids (Fig 3).

Mendonça et al. [27] analyzed cytogenetically hybrids from interspecific crosses between *T*. *lenti* and *T*. *sherlocki* and, as reported in the present study, also observed genomic compatibility between the species (100% pairing between homeologous chromosomes of the hybrids in metaphase I). Riley [68] points out that two species have different genomes when their chromosomes are different so that there is no pairing between one or more pairs of homeologous during hybrid meiosis I, which results in sterility (reproductive isolation) and, consequently, genetic isolation between species (absence of gene flow). However, we suggest that the interspecific genomic compatibility observed, together with absence of pre-zygotic reproductive barrier (Table 2) come from the recent diversification of the taxa of this group, which does not unviable the specific status of the species of this complex (since all species of the *T*. *brasiliensis* complex present genetic distance to the Cyt B mitochondrial gene greater than 2% [28, 39, 40]).

All species and subspecies of the *T*. *brasiliensis* complex share the same chromosomal characteristics [15, 28, 34–38, 69]. Costa et al. [70] suggested that *T*. *b*. *macromelasoma* resulted from the natural crossing between *T*. *b*. *brasiliensis* and *T*. *juazeirensis* by the phenomenon of homoploidal hybridization. Recently, Costa et al. [71], through phenotypic and genotypic analyzes suggested the existence of a natural hybridization zone where interspecific crossings may occur between *T*. *b*. *brasiliensis* and *T*. *juazeirensis*, which supports the origin of *T*. *b*. *macromelasoma* by homoploidal evolution. Natural hybridization events can be more common than previously thought: recently Antunes et al. [72] demonstrated that the choice for the copula is not always towards conspecific females (which may result in increased genotypic and phenotypic variability).

Although *T*. *b*. *macromelasoma* is considered as an incipient species, the low interspecific genetic distance observed between *T*. *b*. *macromelasoma*, *T*. *b*. *brasiliensis* and *T*. *juazeirensis* [69] together with the production capacity of experimental hybrids (Table 2), point to the possibility of natural hybridization events followed by introgression during the evolution of these taxa [69] (which highlights the need for attention of the vector programs with *T*. *juazeirensis*–mainly in view of the recent catches of this species infected with *T*. *cruzi* in human dwellings [73]–(since genes associated with the high vector capacity of *T*. *b*. *brasiliensis* can be fixed in this species by introgression) and with its possible hybrids (that thus the hybrids resulting from the cross between *T*. *sherlocki* and *T*. *juazeirensis* [33] can present higher fitness than their parents).

**Fig 4:**
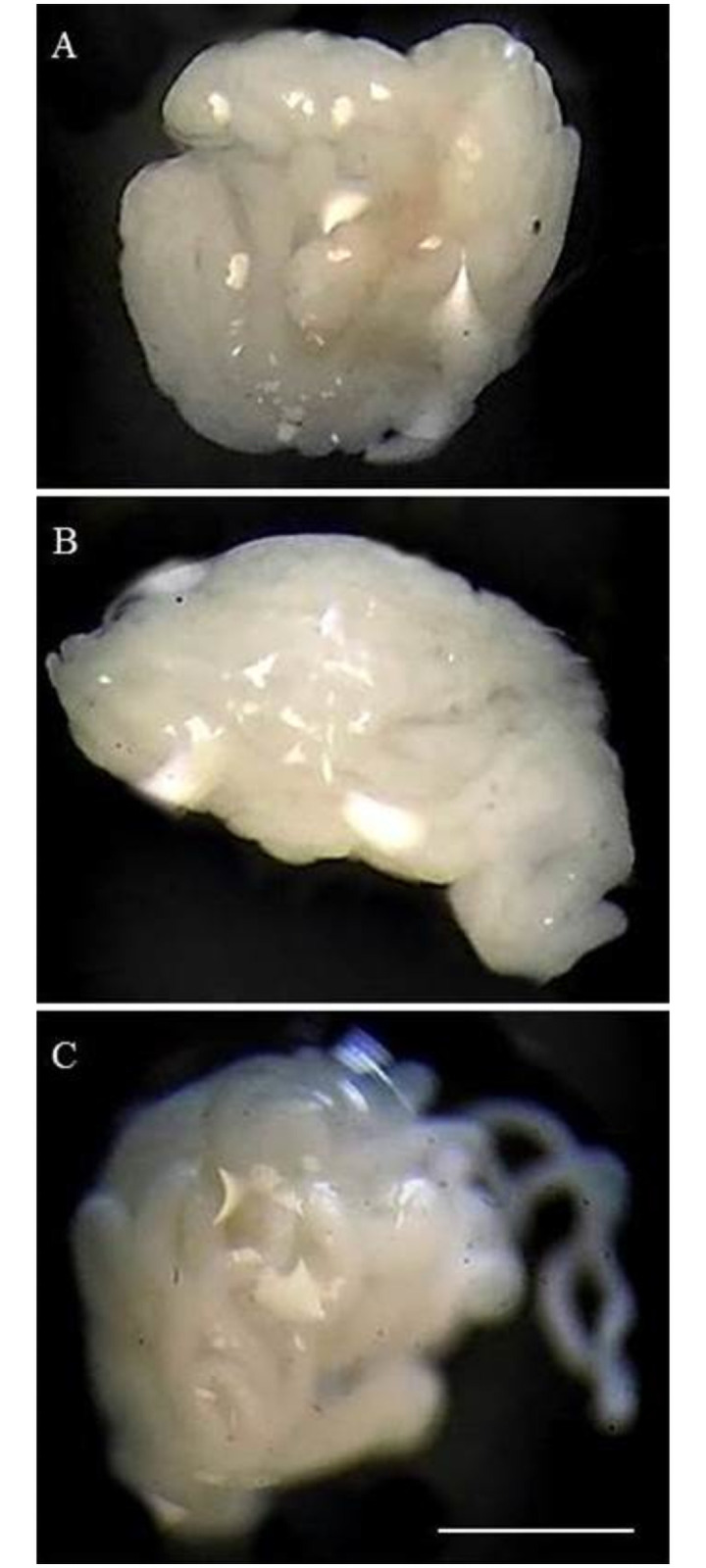
Testicles of adult male hybrids from the cross between *T*. *b*. *macromelasoma* female x *T*. *lenti* male (A), *T*. *juazeirensis* female x *T*. *lenti* male (B) and *T*. *melanica* female x *T*. *lenti* male (C). Note that the gonads do not have gonadal dysgenesis. Bar: 10 mm.

None of the male hybrids analyzed presented the phenomenon of gonadal dysgenesis (Fig 4), that is, the testicles were normal (without atrophy) when compared to the morphology of the testis of Triatominae [74]. This result can be confirmed by the birth of hybrids in all directions of the crossings (Table 1), which demonstrates that both gonads and gametogenesis are normally occurring in first generation hybrids (F1). Gonadal dysgenesis, a phenomenon quite common in drosophilids [75], has never been reported in the subfamily Triatominae. On the other hand, the sterility of the hybrid from the abnormal gametogenesis (chromosomal pairing errors) has been observed several times in these vectors [58, 76, 77].

Although the crosses between *T*. *b*. *brasiliensis* female and *T*. *petrocchiae* male performed by Espínola [59] did not result in hybrids, it is worth mentioning that this author reported hatching of a nymph resulting from the cross between *T*. *petrocchiae* female and *T*. *b*. *brasiliensis* male (Table 2). This result demonstrates that there is still genomic compatibility between these two taxa (even if it’s extremely low), which corroborates the sharing of common ancestry between *T*. *petrocchiae* and species and subspecies of the *T*. *brasiliensis* complex [22], even though *T*. *petrocchiae* is the most distant species of the group, possibly being the taxon with more derived genetic and genomic characteristics.

Recently, was observed that reptile blood is the main food source for *T*. *petrocchiae* [78]. On the other hand, *T*. *b*. *brasiliensis* is mainly associated with rodents [49]. Taking into account that these two taxa live in sympathy [50], it is likely that the ecological (or habitat) isolation is the main pre-zygotic barrier that makes unfeasible the formation of natural hybrids between *T*. *petrocchiae* and *T*. *b*. *brasiliensis*.

Alevi et al. [29] observed pairing error between a pair of homeologous chromosomes of hybrids from the cross between *T*. *bahiensis* and *T*. *lenti*, which highlighted the specific status of *T*. *bahiensis* (for resulting in unviable gametes). The incompatibility of a pair of chromosomes was extremely important from a taxonomic point of view [79]. However, these results emphasize the evolutionary relationship between these species belonging to the *T*. *brasiliensis* complex by the pairing of 90% of the autosomes of these vectors.

## Conclusions

Thus, we demonstrate that there are no pre-zygotic reproductive barriers installed between *T*. *lenti* and the species and subspecies of the *T*. *brasiliensis* complex. In addition, we demonstrate that the hybrids obtained between these crosses have high genomic compatibility (100% pairing between homeologous chromosomes) and the absence of gonadal dysgenesis. These results point to reproductive compatibility between *T*. *lenti* and species and subspecies of the *T*. *brasiliensis* complex (confirming their inclusion in the complex) and lead us to suggest a possible recent diversification of the taxa of this monophyletic group.
